# Multi-story Parkin

**DOI:** 10.18632/oncotarget.18318

**Published:** 2017-05-31

**Authors:** Aitor Martinez, Ugo Mayor, Michael J. Clague

**Affiliations:** Cellular and Molecular Physiology, University of Liverpool, Liverpool, UK

**Keywords:** Parkin, Parkinson’s Disease, cancer, proteomics

Rapid progress has been made in obtaining an inventory of Parkinson’s Disease (PD) associated genes. The challenge now is to understand their interactions and on which physiological pathways they converge. Two major themes have emerged; influence upon mitochondrial dynamics and on trafficking within the endocytic pathway [1]. Parkin (PARK2) is a ubiquitin E3-ligase, for which loss of function mutations lead to PD. Under basal conditions Parkin is largely held in an inactive state. It is recruited to mitochondria by another PD linked protein, the kinase PINK1, which specifically accumulates on depolarised mitochondria. Parkin then promotes mitophagy by ubiquitylating multiple mitochondrial proteins [2]. The rapid and dazzling progress in understanding this coupled recruitment and phosphorylation-dependent activation process has understandably taken centre stage. However, there remains no formal proof that defects in mitophagy lead to PD. Most cellular studies of mitophagy rely upon Parkin over-expression. Irrespective of this matter, it has become clear that Parkin may play more widespread roles that are associated with alternative pathophysiology.

Broader links between Parkin and mitochondrial health have been made in both cardiac maturation and following myocardial infarction [3]. Parkin knock-out mice placed on a high fat diet resist weight gain, steatohepatitis and insulin resistance. They also show increased susceptibility to Tuberculosis infection through a defect in xenophagy. Parkin is also an important tumour suppressor. Decreased PARK2 mRNA or focal deletions are found in more than a third of human cancers. This has been attributed to diminished ATP levels and increased ROS production leading indirectly to inactivation of PTEN [4]. Mutations in the same amino acid are found in cancer and PD.

Discovery-based proteomic approaches offer the opportunity to identify E3-ligase substrates in an unbiased manner. Quantitation of protein levels may reflect differences in turnover rate but can also report indirect effects on transcription/translation. Alternatively ubiquitylated peptides can be directly detected by a signature diGly modification following trypsinisation. We have recently used an alternative method (BioUb) whereby a tagged form of ubiquitin is stably expressed and biotinylated *in vivo*. Ubiquitylated proteins from mice, flies or human cells can then be isolated by Neutravidin beads under stringent washing conditions. This provides greater peptide coverage of substrates although the actual site of ubiquitylation is not always identified [5].

Acute mitochondrial depolarisation provides the only known trigger for Parkin activation and therefore offers a means to facilitate the identification of substrates. Using this procedure in tissue cell culture models has revealed a broad swathe of mitochondrial substrates, with some bias towards modification with ubiquitin chains linked through Lys6 [6]. The overall picture is that a distributed ubiquitin signal across the mitochondrial surface comprised of many substrate proteins is sufficient for Parkin-dependent mitophagy. There remains a need to identify Parkin substrates under more physiological conditions. Comparative proteomic studies of Parkin -/- vs wild type mice and fly brains have reported some differentially expressed proteins. However, these reports largely pre-date a step change in proteomics technology, that increases protein coverage by an order of magnitude. We have recently used the BioUb method to search for Parkin substrates in a fly model, that compares neuronal cells expressing either wild type Parkin or a ligase dead mutant [7]. Our results come with the caveat of over-expression but do not rely upon any acute drug treatments. Of >1000 proteins that were quantitated, 35 were identified as potential Parkin substrates. Some of these were mitochondrial proteins that had been identified in previous studies, whilst others have been associated with the endosomal pathway, including multiple subunits of the vacuolar ATPase and components of the Endosomal Sorting Complex Required for Transport (ESCRT) machinery, ALiX and Vps4. One substrate, the retromer component Vps35, mediates trafficking from endosomal compartments to the Golgi and the plasma membrane. It is itself a PD associated gene that shows genetic interaction with Parkin in a fly model [8].

**Figure 1:**
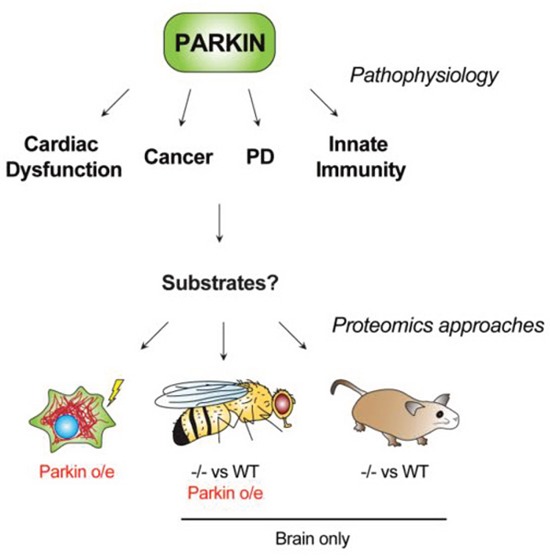
Pathophysiologies relevant to Parkin function and unbiased proteomic strategies aimed at substrate identification in different systems

Interest in Parkin function is clearly broadening and unbiased proteomics screens offer opportunities to open up new biology. Methods have been developed to detect direct substrates and advances in label-free proteomics offer opportunities for tissue wide analyses. Looking forward, these might have less specific emphasis on the brain as the relevance to other types of pathophysiology is now clear.
